# Using the cognitive rigidity-flexibility dimension to deepen our understanding of the autism spectrum

**DOI:** 10.1017/pen.2025.10002

**Published:** 2025-09-01

**Authors:** Shannon Cahalan, Stephen R. Mitroff, Francys Subiaul, Gabriela Rosenblau

**Affiliations:** 1 Department of Psychological and Brain Sciences, The George Washington University, Washington, DC, USA; 2 The GW Autism and Neurodevelopmental Disorders Institute, Washington, DC, USA; 3 Department of Speech, Language and Hearing Sciences, The George Washington University, Washington, DC, USA; 4 Center for the Advanced Study of Human Paleobiology, The George Washington University, Washington, DC, USA

**Keywords:** autism spectrum disorders, cognitive flexibility, dimensional classification

## Abstract

Autism Spectrum Disorder (ASD) is defined as a unidimensional condition, and autism traits are measured on a continuum where the high end of the spectrum represents individuals likely to have an ASD diagnosis. However, the large heterogeneity of ASD has thrown this unidimensional conceptualization into question. With the exact underlying cause(s) of autism yet to be identified, there is a pressing need to establish core, underlying dimensions of ASD that can capture heterogeneity within the autism spectrum, thereby better specifying both autistic traits and ASD symptoms. Here we describe one important transdiagnostic dimension, *the cognitive rigidity-flexibility dimension*, that may impact autistic traits and symptoms across symptom-relevant cognitive domains. We first discuss how diminished cognitive flexibility manifests in core autistic traits and autism symptoms in perception, attention, learning, social cognition, and communication. We then propose to supplement assessments of autistic traits in the general population and autism symptoms in individuals with an ASD diagnosis with a comprehensive batter of cognitive flexibility measures in these symptom-relevant domains. We conjecture that systematic differences in domain-general versus domain-specific cognitive flexibility can distill subgroups within the autism phenotype. While we focus on the cognitive flexibility dimension here, we believe that it is important to extend this framework to other higher order dimensions that can capture core autism symptoms and transdiagnostic symptom severity. This approach can characterize the latent, multi-faceted structure of autism, thereby yielding greater precision in diagnostic classification and the creation of more targeted interventions.

## 1. Introduction

Autism Spectrum Disorder (ASD) is a neurodevelopmental disorder characterized by a combination of social deficits such as atypical social interactions, including poor nonverbal social communication (e.g., reduced eye-contact, emotional expression); difficulty initiating and maintaining interpersonal relationships (e.g., friendships); and the presence of repetitive behaviors (e.g., excessive, atypical fidgeting) and restricted interests (e.g., obsessive/compulsive thoughts, American Psychiatric Association, 2013). While diagnostic criteria necessitates the presence of both social deficits and restricted and repetitive behaviors (RRBIs, American Psychiatric Association, 2013), it is thought that the *quality* of autism itself is unidimensional, even if the *quantity* of core characteristics varies across the spectrum. That is, autism is a single condition with uniform etiology of graded severity (Kamp-Becker et al., 2010; Lundström et al., 2012; Ring et al., 2008). This conceptualization of autism coincides with the idea of a “broader autism phenotype” in which clinically significant autistic traits represent the extreme end of a distribution of traits that are also present in the general population (Austin, 2005; Baron-Cohen et al., 2001; De Groot & Van Strien, 2017; Lundström et al., 2012). This unidimensional view of autism may overlook its complexity. We propose a multidimensional approach to account for the variability in autism presentations, focusing on cognitive rigidity as a key factor. We detail how cognitive rigidity can underlie differences in attention, learning, social cognition, and language in autism and propose an approach to clarify whether cognitive flexibility can be considered one dimension across these domains or whether it is itself a multidimensional construct.

There is growing evidence challenging the idea of a unidimensional autism phenotype, however (Beglinger & Smith, 2001; Hong et al., 2018; Mottron & Bzdok, 2020; Ousley & Cermak, 2014). The types of social deficits and RRBIs vary widely among individuals with ASD^1^. Classifying these core dimensions as either present or absent may mask more fine-grained differences between individuals which have broader impacts on adaptive functioning (Bone et al., 2016; Happé & Ronald, 2008; Mottron & Bzdok, 2020; Ousley & Cermak, 2014). Furthermore, nearly three-quarters of individuals with an autism diagnosis have a co-occurring condition that interacts with or contributes to the presentation of autistic traits (Joshi et al., 2010; Khachadourian et al., 2023; Mannion & Leader, 2013; Masi et al., 2017; Ousley & Cermak, 2014; Sauer et al., 2021; Shoaib et al., 2022). While comorbidities add heterogeneity and challenges for specifying autism diagnosis and interventions, common co-occurrences can offer insight into potential symptom clusters, which align with a multidimensional view (Agelink Van Rentergem et al., 2021; Hong et al., 2020; Mottron & Bzdok, 2020; Ousley & Cermak, 2014; Schwartzman, 2016).

Phenotypic heterogeneity of ASD is mirrored on the genetic and neurobiological levels as well (Betancur, 2011; Ecker, 2017; Geschwind & State, 2015; Hong et al., 2018; Sauer et al., 2021; Shan et al., 2022; Warrier et al., 2022). Genetically defined conditions like Fragile X Syndrome, Rett Syndrome, or tuberous sclerosis complex, account for approximately one to two percent of autism cases (e.g., Caglayan, 2010; Sztainberg & Zoghbi, 2016). The vast majority of ASD diagnoses, however, are nonsyndromic and linked to mutations in about 100 genes with compounding effects on autism phenotypes (Betancur, 2011; Caglayan, 2010; Geschwind & State, 2015; Rolland et al., 2023; Schaaf & Zoghbi, 2011; M. J. Taylor et al., 2020; Warrier et al., 2022). Some genetic variants may result in certain features of autism, but there is no conclusive evidence of specific genetic profiles producing phenotypic subtypes (Happé & Ronald, 2008; Rolland et al., 2023; Warrier et al., 2022). Ultimately, tracing behavioral profiles to genetics and neurobiology has yet to yield any precise differentiation of autism phenotypes (Geschwind & State, 2015; Rolland et al., 2023; M. J. Taylor et al., 2020; Volkmar et al., 2009; Warrier et al., 2022).

The heterogeneity of ASD has posed significant challenges for developing targeted interventions (Chen & Geschwind, 2022; Ghosh et al., 2013; Green & Garg, 2018; Lombardo et al., 2019; Masi et al., 2017; Sauer et al., 2021), a difficulty that extends to the diagnosis of neuropsychiatric disorders more broadly. In response, there have been growing calls for more innovative and multidimensional approaches to classification and intervention (Cuthbert, 2005, 2014; Krueger et al., 2018; S. E. Morris et al., 2022). Rather than establishing whether a neuropsychiatric condition is present or absent – as is the case in the current categorical model – conditions are conceptualized as symptom clusters associated with maladaptive, observable behaviors across disorders. Thus, research would shift to finding the genetic and neurobiological roots of specific symptom clusters and developing targeted treatments and interventions which can bolster individualized treatment plans across conditions (American Psychiatric Association, 2013; Cuthbert, 2014; World Health Organization, 1992). Krueger and colleagues (2018) formalized an alternative, multidimensional approach by introducing the Hierarchical Organizational Structure of Psychopathological Dimensions (HiToP). The HiToP proposes a focus on transdiagnostic, continuous, higher-order dimensions (i.e., internalizing behaviors), which can be decomposed into frequently co-occurring subdomains (i.e., sleep distress, eating pathology, nervousness, and irritability). These more specific subdomains can then guide classification into diagnoses like depression or anxiety (Krueger et al., 2018; Michelini et al., 2021, 2024). Recently, Chetcuti et al. (2025) proposed the HiToP as a useful framework for conceptualizing autism as multidimensional.

This multidimensional conceptualization of autism could not only constrain the heterogeneity of the autism phenotype (Agelink Van Rentergem et al., 2021; Beglinger & Smith, 2001; Chetcuti et al., 2025; Geschwind & Levitt, 2007; Hong et al., 2020; Ousley & Cermak, 2014; Schwartzman, 2016) but also guide research into the genetic causes of autism (Beversdorf & Consortium*, 2016; Hong et al., 2020; Mottron & Bzdok, 2020; Ousley & Cermak, 2014). In order to probe whether autism is multidimensional, there needs to be efforts to acquire larger sample sizes, broader institution of finer-grained assessments of core autistic traits, and implementation of multivariate analytic techniques to derive relevant dimensions.

The National Institute of Health (NIH) and the Simons Foundation for Autism Research have identified maximizing sample size as a key funding priority for autism research. The Autism Centers of Excellence program funded by the NIH serves as a multisite research consortium aiming to adopt consistent methodology across sites and consolidate and archive research data sets via the National Institute of Mental Health Database for Autism Research (https://nda.nih.gov). Likewise, the Simons Foundation launched the Simons Foundation Autism Research Initiative in order to acquire larger, robustly characterized datasets of autistic individuals (Feliciano et al., 2018). These are promising initiatives for acquiring sample sizes large enough to thoroughly characterize potential subgroups of diagnosed individuals with the goal of determining relevant dimensions that stratify autism phenotypes. Nonetheless, it is important not only to focus on individuals with an ASD diagnosis, but also study autistic traits in the general population in order to assess convergence with clinically significant symptoms.

To this end, we suggest specifying *the cognitive rigidity-flexibility dimension* as one candidate higher-order dimension since it relates to autistic traits in the general population (Gökçen et al., 2014; Goris et al., 2020) and is linked to autism symptom load in individuals with an ASD diagnosis (Hollocks et al., 2022; Lage et al., 2024; Mostert-Kerckhoffs et al., 2015). In the following sections, we also elaborate on how *the cognitive rigidity-flexibility dimension* is currently defined and measured and how it relates to patterns of perception, attention, learning, social cognition, and language in individuals with a diagnosis of ASD. Examining functioning across these subdomains as it relates to cognitive flexibility can aid in clustering phenotypes. We also propose an assessment framework based around the *cognitive rigidity-flexibility dimension* in these cognitive subdomains. This framework would support investigations of the convergence between autistic traits in undiagnosed individuals and in those with an autism diagnosis and support multivariate analyses aimed at distilling potential subgroupings in a multidimensional space.

## 2. The latent structure of autism: uni- versus multi-dimensional perspectives

There has been significant debate regarding the latent structure of autism: whether it is uni- or multi-dimensional. The unidimensional perspective asserts that autistic traits span a continuum with the extreme end of the distribution comprising individuals that can be diagnosed with autism based on core deficits in social communication and reciprocity and RRBIs (Baron-Cohen et al., 2001; Kamp-Becker et al., 2010; Lundström et al., 2012; Ring et al., 2008). A number of studies argue that the distribution of autistic traits in the general population and in individuals with a diagnosis are separable, however (Abu-Akel et al., 2019; Wittkopf et al., 2023). Moreover, Mottron and Bzdok (2020) argued that without a clear etiology for ASD, it remains uncertain whether autistic traits in the general population emerge from the same underlying cause as symptoms of ASD.

As discussed, addressing this challenge will necessitate thoroughly characterizing large samples of undiagnosed and diagnosed individuals. Limiting samples to a specific diagnostic status introduces an issue of circularity (Abu-Akel et al., 2019; Happé & Frith, 2020). Sampling solely from individuals meeting current diagnostic criteria for autism may artificially constrain characterization of potential subgroups to the status quo. An increasing number of individuals with ASD receive a late diagnosis because they present with autistic traits that are less consistent with current diagnostic criteria (D’Mello et al., 2022; Harrison et al., 2017; Loomes et al., 2017). For example, compared to individuals diagnosed in early childhood, late-diagnosed individuals present with more social deficits. In contrast, early diagnosed individuals reported more communication deficits and RRBIs (Bone et al., 2016; Wallace et al., 2017). Restricting phenotyping efforts to individuals with an ASD diagnosis also primarily benefits males, as boys are much more frequently diagnosed than girls. Autistic females are also less likely to receive a proper and timely diagnosis because they show greater social engagement and communication abilities and fewer repetitive behaviors than their male counterparts (Bargiela et al., 2016; Corbett et al., 2021; Ferri et al., 2018; Hiller et al., 2016; Sedgewick et al., 2016; Whitlock et al., 2020). Self-identified autistic individuals are also often excluded from research to maintain rigor in sampling and ensure that there is a level of standardization among autistic samples. However, including self-identified individuals may improve phenotyping efforts. On one hand, self-identified individuals may comprise a subgroup who, compared to those with a diagnosis or meeting current diagnostic thresholds, are distinct. As a comparison group to clinically diagnosed people, their inclusion can improve diagnostic specification and highlight similarities and differences between these groups. More importantly, self-identified autistic individuals may not be distinct phenotypically from other autistic individuals but rather face structural and socioeconomic barriers to accessing clinical diagnosis. In turn, including this groups would shed light on both potential current diagnostic limitations, as well as, how diagnostic access is shaped by structural barriers, stigma, and clinician biases (Ardeleanu et al., 2024; Lewis, 2017; Lockwood Estrin et al., 2021; Overton et al., 2024).

However, including large samples of diagnosed and undiagnosed individuals would only determine whether the autism spectrum is composed of a single, continuous distribution comprising both the general population and those meeting clinical criteria for autism or separable distributions. Another issue posed by the unidimensional perspective is that conceptualizing autistic traits as a composite, single dimension fails to capture variability across subdomains, which – in turn – would allow the exploration of subgroups. Multiple studies have found only modest correlations between subscale scores of the Autism Quotient (AQ, Baron-Cohen et al., 2001) and have debated the five-factor structure and suggested the scale is comprised of less factors (English et al., 2020; Hoekstra et al., 2008). For example, attention to detail shows modest correlations with social skills, communication, and imagination domains, which are often more intercorrelated, suggesting attention to detail captures a distinct feature of the autism phenotype. Moreover, the same sum score on the AQ does not mean that individuals have the same constellation of autistic traits with respect to its subdomains (English et al., 2020). One participant may have endorsed mostly items pertaining to attention to detail while another may have endorsed items across all sub-factors. English and colleagues (2020) proposed that heterogeneity in AQ subfactor loadings within the general population offers further support for a multidimensional view of autism and potential subtypes under the umbrella of the autism phenotype.

Additionally, self-report measures, such as the AQ, have mixed validity when applied to the general population versus those with a diagnosis of ASD. Alexithymia and difficulties with introspective thinking are often reported in autism, rendering self-reports more unreliable in this population (Gaigg, Cornell & Bird, 2018; Kinnaird et al., 2019; Mazefsky et al., 2011; Sizoo et al., 2015). Examining convergence between informant and self-reports is one avenue toward a more objective account of autistic traits in individuals diagnosed with ASD.

Incorporating experimental paradigms capturing functioning in cognitive subdomains relevant to autism symptomatology – such as perception and attention, social cognition, and language – could further circumvent issues with objective reporting of symptoms or traits. Crucially, integrating experimental tasks in this way could facilitate the application of multivariate, data-driven analysis to arbitrate whether a single or multiple dimensions capture meaningful variability in autistic traits of symptom profiles. To this end, novel dimensionality reduction techniques, like exploratory graph analysis (EGA, Golino & Epskamp, 2017), are particularly well-suited for deriving and visualizing the latent dimensions underpinning large, multimodal, and potentially noisy and heterogeneous data (Golino et al., 2020). Confirmatory factor analysis can also test the validity and stability of EGA-derived dimensions across different populations (Brown, 2015). If multiple dimensions can be derived based on task-based performance in relevant cognitive domains, then predictive modeling with more traditional assessments, like the AQ, could determine whether the dimensions capture meaningful differences in autistic profiles and not just noise. Techniques like latent profile analysis and Gaussian Mixture Modeling can also be applied for defining behaviorally and functionally relevant symptom profile clusters based on variability across multiple dimensions (Oberski, 2016; Scrucca et al., 2016).

For brevity, we focus on specifying one latent dimension, the cognitive rigidity-flexibility dimension, across various cognitive subdomains relevant to ASD. The cognitive rigidity-flexibility dimension can be probed objectively across both nonautistic and autistic populations through experimental tasks in various cognitive domains. More importantly, cognitive flexibility as it manifests in cognitive subdomains is a key precursor to both core symptom clusters: RRBIs (Mostert-Kerckhoffs et al., 2015; Pugliese et al., 2015; South et al., 2007) and social reciprocity and communication (Bertollo et al., 2020; Costescu et al., 2022; Rosenblau et al., 2023). Currently, this dimension has been somewhat neglected as a domain-general process facilitating key features of social cognition in ASD (Geurts et al., 2009; Kissine, 2012; Rosenblau et al., 2023). Yet, interventions aiming to improve cognitive flexibility in autistic individuals not only improve RRBIs but also social skills (Bertollo et al., 2020; Kenworthy et al., 2014).

Here we focus on the rigidity-flexibility dimension as emerging studies find that it is a meaningful predictor of social and communication skills of autistic individuals – which have been traditionally deemed to be independent from rigidity in the context of RRBIs (Bertelsen et al., 2021; Happé & Ronald, 2008; Rosenblau et al., 2023). There is theoretical support, however, for other transdiagnostic dimensions which underpin defining characteristics of ASD. Such examples include sensory reactivity (Williams et al., 2023), personality and temperament (Burrows et al., 2016; Lodi-Smith et al., 2019; Schwartzman, 2016), and internalizing versus externalizing behaviors (Bauminger et al., 2010; Rodriguez-Seijas et al., 2020). Our recommendations for understanding the role of cognitive rigidity in subdomains relevant to autism can be extended to these other relevant dimensions, studied separately or in combination.

## 3. What is cognitive flexibility and how has it traditionally been studied?

Cognitive flexibility refers to one’s ability to recognize changes in the environment and efficiently adapt to these changes (Denckla, 1996; Ionescu, 2012; Monsell, 2003). Cognitive flexibility supports adaptive living skills in everyday life, including managing change and transitions in routines and schedules; monitoring and adjusting actions to meet responsibilities; and facilitating appropriate social communication and interaction (Hollocks et al., 2022; Ionescu, 2012; L. Morris & Mansell, 2018; Rosenblau et al., 2023). For example, parents exhibit cognitive flexibility daily when they adapt their parenting strategies to accommodate the evolving needs of a growing child. Children change and reverse personal preferences rapidly and abruptly. For instance, a child might insist on wearing certain clothes, eating specific foods, or playing with particular toys, only to later show no interest or develop an active dislike for these items. Parents, in turn, must recognize these shifts, which may be communicated implicitly or explicitly. Cognitive flexibility allows parents to adjust their approach accordingly to support the child’s changing preferences and developmental needs.

In autism, cognitive flexibility is strongly associated with RRBIs, particularly an “insistence on sameness” (Bodfish et al., 2000; D’Cruz et al., 2013; Lopez et al., 2005; Mostert-Kerckhoffs et al., 2015; Yerys et al., 2009). It also predicts socialization and adaptive living skills in autism as measured by the Vineland Adaptive Behaviors Scale (Bertollo et al., 2020; Pugliese et al., 2015, Wallace et al., 2016). This is particularly evident during the transition to adulthood, which offers unique challenges for those diagnosed with autism as the predictable routines of childhood are disrupted and individuals learn to foster independence (Biggs & Carter, 2016; Wallace et al., 2016). Irrespective of intellectual ability, cognitive rigidity in this transition period poses challenges to individuals’ independence in vocational and social settings.

Traditional paradigms that study cognitive flexibility in isolation typically involve matching items to a target based on a relevant features and then switching the relevant feature in question to measure how fast participants notice and adapt to the change (e.g., Denckla, 1996; Van Eylen et al., 2011). A prominent example of this type of task is the Wisconsin Card Sorting Test (WCST, Grant & Berg, 1948). In the WCST, participants are presented with a target and two items matching the target’s color or shape. The participant must select the correct item either by matching it based on shape or color to the target. This means that participants must implicitly infer the matching rule based on task feedback. Flexibility is measured through perseverative errors and switch costs in reaction time (RT). Perseverative errors occur when the participant continually applies the prior rule for several trials after a rule switch. Switch-costs are described as higher RTs relative to trials before the switch in subsequent trials after the switch. A more flexible participant who adapts to the changing rules would produce fewer perseverative errors and have overall lower RTs, especially following a switch.

The WCST assesses cognitive flexibility through the mechanism of implicit associative learning, also referred to as reversal learning (Dias et al., 1997; Monni et al., 2023; Wildes et al., 2014; Yerys et al., 2009). Reversal learning tasks, which will be broached further in a subsequent section, measure the ability of individuals to adapt to ever changing, and in some instances reversing, contingencies in the environment – an important skill in everyday life. Reflecting cognitive flexibility’s integrated role in executive functions, on the neural level, cognitive flexibility tasks recruit a distributed network of frontoparietal regions including the inferior frontal junction, ventrolateral prefrontal cortex, posterior parietal cortices, insula, and anterior cingulate cortices (Dajani & Uddin, 2015; Kim et al., 2012; Niendam et al., 2012).

The WCST has been used to differentiate between individuals with and without an autism diagnosis. Participants with an autism diagnosis produce more perseverative errors and higher switch costs compared to controls indicating higher levels of cognitive rigidity (Geurts et al., 2009; Leung & Zakzanis, 2014; Van Eylen et al., 2011). While these findings offer compelling evidence for the WCST capturing cognitive rigidity in autism, Geurts and colleagues (2009) caution that cognitive rigidity, as measured with the WCST, needs to be related back to behavioral rigidity observed in everyday life. While there is a correlation between performance in the WCST and repetitive and restricted behaviors in autism, which may be the most overt expressions of behavioral rigidity (South et al., 2007), limited studies specifically examine how WCST performance translates to difficulty adapting among individuals with ASD to the changing environment in daily life. Nonetheless, behavioral rigidity, in the form of difficulty adapting to changes in daily life, is widely reported in autism and is predictive of adaptive functioning, outcomes, and psychological well-being in autistic individuals (Bertollo et al., 2020; Kraper et al., 2017; Pugliese et al., 2015; Wallace et al., 2016).

High levels of rigidity in autism are evident in a wide range of domains from daily routines and rituals to personal preferences (Baraskewich et al., 2021; Byrska et al., 2023; Faja & Nelson Darling, 2019; Spackman et al., 2025). Cognitive rigidity in terms of shifting perspectives to adopt or infer the perspectives of others have been closely tied to the social deficits of individuals with ASD (Bertollo et al., 2020; C. Frith & Frith, 2005; Geurts et al., 2009; Rosenblau et al., 2015, 2023).

**Figure 1.**
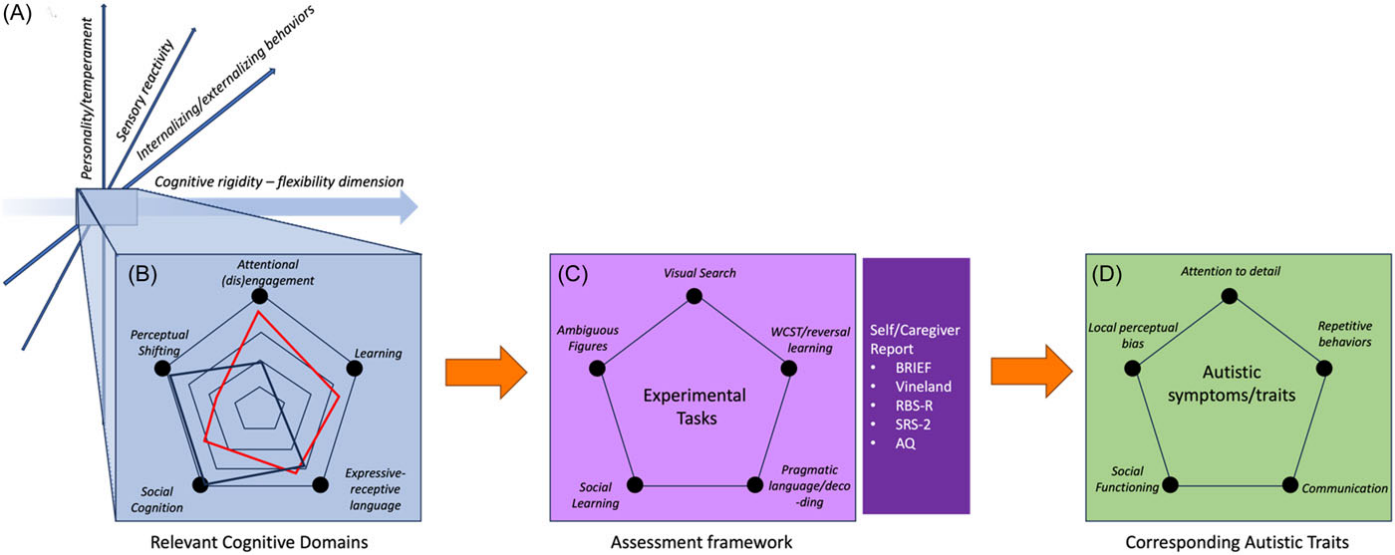
A framework for specificying autism phenotypes based on the cognitive rigidity-flexibility dimension. Panel A) Transdiagnostic higher order dimensions which may underpin core behavioral domains in ASD. Panel B) Cognitive functions or sub-domains relevant to ASD which are supported by cognitive flexibility. Individual profiles of various individuals with autism (in red and black) may meaningfully differ in these cognitive domains relevant to autism. Panel C) An assessment framework incorporating experimental tasks which tap into cognitive flexibility as it manifests in each cognitive domain. This would complement traditional self-/informant report measures used for autism diagnosis and characterization including the Autism Quotient (AQ), Social Responsiveness Scale - 2nd Edition (SRS-2), The Broad Inventory of Executive Functions (BRIEF), Vineland Adaptive Behaviors Scale, Repetitive Behaviors Scale - Revised (RBS-R). Panel D) Using this framework, multivariate analyses can assess how cognitive flexibility across these domains maps onto core trait domains of the autism phenotype.

In summary, cognitive flexibility has wide-reaching implications for learning and refining adaptive living skills in the real world. Evidence suggests that greater cognitive rigidity, as opposed to flexibility, in autism may account for difficulties with adaptive living skills; most notably, the transitions and uncertainties individuals face in daily life. In the following section, we detail how cognitive flexibility is reflected in various subdomains of autism symptomatology such as perception, attention, learning, communication, and social inferences (See Figure 1, Panel B).

## 4. Cognitive rigidity is a hallmark of autism across various cognitive domains

While the core diagnostic dimensions for autism, that is, RRBIs and social deficits, have been proposed as separable (Happé & Ronald, 2008), recent evidence suggests cognitive flexibility may facilitate both RRBIs and deficits in social cognition. Several studies have shown that performance in experimental paradigms probing cognitive flexibility, like the WCST, predict RRBIs (D’Cruz et al., 2013; South et al., 2007, 2012; Yerys et al., 2009). Most studies that show a link between cognitive flexibility and social functioning in autism have relied on self- or parent-report measures only (Bertollo et al., 2020; Gioia et al., 2000; Pugliese et al., 2015). One experimental study, however, showed a more direct influence of cognitive flexibility and social functioning. An intervention that was developed to remediate children’s repetitive and rigid behaviors translated to improvements in their social functioning (Kenworthy et al., 2014).

Based on this emerging evidence, we propose that the cognitive rigidity-flexibility spectrum may represent a crucial latent dimension influencing multiple symptom domains in autism, including the core dimensions of RRBIs and social deficits. Focusing on this and other latent dimensions within specific cognitive subdomains, can enhance our ability to stratify autism phenotypes within and across symptom domains. This approach could offer more nuanced subclusters of autism, provide insights into the role of individual differences in autism phenotypes, and ultimately spur the development of tailored interventions for individuals on the autism spectrum.

### 4.1. Visual perception and attention

Cognitive flexibility in visual perception manifests in the ability to shift perceptual focus, engage or disengage from salient stimuli, and adapt attentional priorities in response to changing task demands. Paradigms that require cued attention-switching between stimulus features (e.g., Navon Task, Duck–Rabbit illusion) or filtering distractions to detect a target (e.g., visual search) draw on domain-general cognitive flexibility (Geddert & Egner, 2022; Kaldy et al., 2016; Mitroff et al., 2006; Qiao et al., 2020; Wilkinson et al., 2001). Despite, evidence supporting preserved ability to detect low-level visual stimuli and intact visual acuity (Ashwin et al., 2009; Dakin & Frith, 2005; Koldewyn et al., 2010; Van De Cruys et al., 2014), individuals with an autism diagnosis display distinct perceptual biases which cascade to attentional priorities. Likewise, this population exhibits greater resistance to disengaging attention and shifting attentional focus across these paradigms.

People typically show a global processing bias on the Navon task, favoring holistic over local features (Kimchi, 1992; Navon, 1977; Wilkinson et al., 2001). In contrast, individuals with an autism diagnosis tend to prioritize local features as opposed to global features (Happé & Frith, 2006; Mottron et al., 2006; Pellicano et al., 2005). Evidence suggests that reduced influence from top-down executive control regions, including frontoparietal networks implicated in cognitive flexibility, in filtering noisy, bottom-up, sensory input underpin the distinct local bias in ASD (Brock, 2012; Dajani & Uddin, 2015; Friston et al., 2013; Pellicano et al., 2005; Shafritz et al., 2008; Van De Cruys et al., 2014). Critically, these same top-down networks drive the ability to more quickly overcome perceptual biases when cued suggesting cognitive flexibility plays a critical role in the renegotiation of attention depending on task demands (Qiao et al., 2020). In individuals without an autism diagnosis, cognitive flexibility predicts quicker ability to overcome global biases and attend to local features when cued in the Navon task (Brockmeyer et al., 2022; Geddert & Egner, 2022; Navon, 1977). Individuals diagnosed with ASD tend to have higher switch costs when shifting to attend to the global gestalt in the Navon Task and this was likewise positively correlated with their performance in set-shifting paradigms (Richard & Lajiness-O’Neill, 2015; Soriano et al., 2018). A related phenomenon – the ability to spontaneously reverse ambiguous figures like the Duck-Bunny illusion – coincides with the development of frontoparietal networks and likely also leverages cognitive flexibility (Scocchia et al., 2014; Wimmer et al., 2011). As top-down input strengthens in children they are able to more flexibly perceive the duck as opposed to the bunny when cued or vice versa (Mitroff et al., 2006). Mirroring findings in the from the Navon task, children with an autism diagnosis continue to perseverate on either the duck or bunny interpretation of the illusion and report reduced spontaneous reversals when cued (Sobel et al., 2005). Together, evidence from these paradigms offers a coherent narrative wherein both neural and behavioral evidence suggest individuals with an autism diagnosis may persist in their perceptual biases because of reduced influence of goal-oriented processes which facilitate perceptual shifts.

However, persistence of a local perceptual bias in ASD may be advantageous in tasks requiring attention to detail, such as visual search, by enhancing the ability to filter distractors and identify targets, likely due to reduced top-down modulation and resistance to attentional disengagement (Kaldy et al., 2016; Keehn et al., 2013; Landry & Bryson, 2004; Muth et al., 2014; Van De Cruys et al., 2014). Several studies suggest individuals with ASD outperform controls in visual search tasks regardless of the complexity of the search array (e.g., set size, how similar distractors were to the target, and so on; Gregory & Plaisted-Grant, 2016; Kaldy et al., 2016; Sabatino DiCriscio & Troiani, 2017). Critically, the visual search advantage associated with autism is not invariable and contextual factors seem to modulate autistic search performance. For example, increasing perceptual load results in stronger distractor interference effects – or a larger discrepancy in reaction times between high and low load trials – among individuals with more autistic traits (Bayliss & Kritikos, 2011). Additionally, autistic individuals did not outperform controls in more naturalistic, search scenes (Russell et al., 2019). A meta-analysis that integrated these contradicting findings concluded that the visual search advantage in autistic people may be in the low to moderate range (Constable et al., 2020).

While more research is needed to more directly link cognitive flexibility with visual search performance in autism, there is evidence to suggest that advantageous attentional perseveration on local features in a search array may stem from reduced influence of top-down regions, implicated in cognitive flexibility, on bottom-up perceptual regions (Kaldy et al., 2016). At the same time, this attenuated influence of higher cognitive processes on perception in ASD may diminish the resolution of perceptual biases when the task at hand necessitates shifting between different features. These examples highlight the role of cognitive flexibility in shaping visual perception and selective attention in autism.

### 4.2. Learning from and adapting to task environments

Adapting to constant environmental changes is essential for survival. Learning means flexibly adjusting both our behavior and our internal models of the world in response to changing contingencies (Behrens et al., 2018; Lynn & Bassett, 2020). Cognitive flexibility is a crucial driver in both maintaining up-to-date representations of statistical regularities in the environment and accommodating and overcoming irregularities (Amso & Davidow, 2012; Behrens et al., 2007, 2018; Feng et al., 2020; Jahn et al., 2024; Lynn & Bassett, 2020). Implicitly tracking and updating our beliefs about the probabilistic relationships between objects, concepts, or events in what is referred to as statistical learning (Vapnik, 1999).

A well-known experimental approach for studying cognitive flexibility, which draws on the concept of statistical learning, is the reversal learning paradigm. In reversal learning tasks, such as the WCST discussed in a previous section, participants are asked to learn a rule that governs the ordering or categorization of various stimuli. After a certain interval, the rule reverses, and participants must detect this shift and quickly adapt to the new contingency. This paradigm effectively captures how learning can drive shifts in cognitive representations of feature relationships to support flexible behavior (Cools et al., 2002; Costa et al., 2015; Izquierdo et al., 2017).

The WCST and similar paradigms have revealed that, compared to controls, individuals with ASD adapt more slowly to new contingencies (Costescu et al., 2015; Yerys et al., 2009; Zalla et al., 2009). In the context of both reward and fear conditioning, individuals with an autism diagnosis adapt more slowly to rule changes compared to controls (D’Cruz et al., 2013; South et al., 2012). Moreover, children with an autism diagnosis showed an altered developmental trajectory in reversal learning compared to children without an autism diagnosis. In nonautistic children, reversal learning performance improved into adolescence while children with ASD showed delayed improvements in reversal learning compared to controls (D’Cruz et al., 2013). Increased regressive errors (i.e., applying an outdated rule) and delayed acquisition of the new rule both predicted RRBIs, particularly difficulties with transitions and the need for routine, in individuals with ASD (D’Cruz et al., 2013; South et al., 2012).

The WCST measures reversal learning performance in two ways: through intra-dimensional and extra-dimensional set shifts. Intradimensional shifting refers to shifts within a feature type (i.e., continuing to match cards by color, but the specific color changes), while extra-dimensional shifting involves shifting the matching rule entirely to a different feature (i.e., switching from a color to a shape matching rule; Monni et al., 2023; Yerys et al., 2009). Both intra- and extra-dimensional shifts require cognitive flexibility, but extra-dimensional shifts are harder, and therefore require a higher level of cognitive flexibility (Oh et al., 2014; Ozonoff et al., 2004; Yerys et al., 2009). This is why extra-dimensional reversals are harder for individuals with an autism diagnosis (Geurts et al., 2009; Ozonoff et al., 2004; Yerys et al., 2009). Slower shifts in extra-dimensional reversal learning has been shown to scale with behavioral inflexibility in individuals with an autism diagnosis (D’Cruz et al., 2013; Geurts et al., 2009; Leung & Zakzanis, 2014; Yerys et al., 2009). The WCST and similar tasks have captured diminished cognitive flexibility in ASD in well-controlled settings. An open challenge is translating these findings to a more complex set of behaviors in daily life, in which there are more statistical irregularities to track, and environmental changes are much less controlled and consistent.

Depending on the task at hand, cognitive flexibility in real life may hinge or interact with other latent cognitive functions, such as processing speed or emotional control, which could diminish or exacerbate the difficulties of individuals with autism. Unprompted changes requiring a larger cognitive load amplify adaptive difficulties in Individuals with an autism diagnosis (Geurts et al., 2009; Van Eylen et al., 2011). Volatility, or how likely contingencies are to shift, is an important factor for how participants adapt to changes (Behrens et al., 2007; O’Reilly, 2013). Moreover, individuals with an autism diagnosis tend to overestimate volatility in the environment, and yet have been shown to adapt learning rates or behavior less to environmental changes (D’Cruz et al., 2016; Goris et al., 2021; Lawson et al., 2017; Sapey-Triomphe et al., 2022; Sevgi et al., 2020). Based on these studies, it can be concluded that individuals with ASD find the world to be noisier and more uncertain than typically developing individuals. The lack of predictability may lead individuals with ASD to the conclusion that environmental feedback is unreliable, and that it is better to stick to their already established expectations or strategies (Lawson et al., 2017; Robic et al., 2015; Sapey-Triomphe et al., 2022; Van De Cruys et al., 2014). Thus, cognitive rigidity in ASD may serve as a compensatory strategy in some respects but have compounding effects on learning and knowledge structures.

### 4.3 Social cognition

Cognitive flexibility supports social cognition, an umbrella term that refers to cognitive processes in social settings (C. D. Frith & Frith, 2008, 2012), by enabling individuals to shift between multimodal social cues and to represent other’s thoughts, feelings, and perspectives which may diverge from one’s own subjective experience. Research suggests cognitive flexibility is a critical precursor to more complex abstractions about others in the social environment (Frith & Frith, 2012; Kloo et al., 2010; Lockwood et al., 2020). Core differences in social cognition in autism may stem from domain-general rigidity and difficulties with shifting.

Social cues in real life are inherently multifaceted and multisensory, incorporating elements such as facial expressions, gestures, tone of voice, and contextual information which implicate a widespread network of neural regions implicated in attention, working memory and cognitive control (Dajani & Uddin, 2015; Frischen et al., 2007; Gross & Medina-DeVilliers, 2020; Haskins et al., 2022; Lockwood et al., 2020; McKinnon & Moscovitch, 2007; Mundy & Newell, 2007; Y-J Yang et al., 2015; Zaki, 2013). Global processing and top-down control in integrating cues is crucial for recognizing someone else’s identity, emotion, or intention through facial cues and biological motion (Barton et al., 2002; Behrmann et al., 2006; Castelli et al., 2002; Farah et al., 1995; Richler et al., 2011). As previously discussed, individuals with an autism diagnosis engage fewer top-down resources in perception resulting in perseverative attention to local features. In turn, individuals with ASD have been shown to process socially relevant stimuli like faces and biological motion less holistically (Behrmann et al., 2006; Bookheimer et al., 2008; Klin et al., 2002; Ristic et al., 2005). These differences in social perception could cascade into difficulty recognizing emotions or inferring intentions (Harms, Martin & Wallace, 2010; McPartland et al., 2011).

Inferring someone else’s point of view requires flexible shifting from one’s own perspective to that of another person. (Batson et al., 1997; C. Frith & Frith, 2006, 2008; C. Frith & Frith, 2005; De Lillo & Ferguson, 2023). Paradigms that probe mentalizing abilities involve identifying complex emotions of peers or subjects in a social interaction (Baron-Cohen et al., 2001; Dziobek et al., 2006; C. D. Frith & Frith, 2006; Pantelis et al., 2015; Rosenblau et al., 2015), or someone else’s false belief – a belief that is based on less information than is accessible to the participant (Hughes et al., 2000; Wellman et al., 2001; H. Wimmer & Perner, 1983). In an intervention study, cognitive flexibility training not only improved children’s performance on set-shifting paradigms, such as the Dimensional Card Sort Task, but also translated to improvements in false-belief measures of mentalizing wherein the children had to represent others’ knowledge (Kloo et al., 2010; Kloo & Perner, 2003). Studies also indicate that, in individuals with an autism diagnosis, better performance in the Dimensional Card Sorting Task predicted the ability to pass false-belief tests (Colvert et al., 2002; Kissine, 2012; Zelazo et al., 2002).

Furthermore, mentalizing engages a widespread network of cortical regions sometimes referred to as the “theory of mind network,” including the temporoparietal regions, medial prefrontal cortex, anterior cingulate cortex, and temporal cortices (C. Frith & Frith, 2006; C. Frith & Frith, 2005; Mar, 2011; Saxe & Kanwisher, 2013; Y-J Yang et al., 2015), several of which also play a crucial role in maintaining cognitive flexibility across various task contexts (Dajani & Uddin, 2015; Lockwood et al., 2020; Ouerchefani et al., 2024; Rosenblau et al., 2023). Among individuals with an autism diagnosis, studies have reported atypical activity and connectivity between regions involved in mentalizing and cognitive flexibility, such as the anterior cingulate cortex and the insula (Dajani & Uddin, 2015; Gotts et al., 2012; Keehn et al., 2013; Sami et al., 2023; Uddin, 2015; Yerys et al., 2015). While both behavioral and neural evidence point to a facilitating role of cognitive flexibility in key social cognition processes like mentalizing, there is a need for more nuanced assessments that quantify the level of cognitive flexibility in social settings. These measures will make an important contribution to developing targeted interventions for autism that enhance cognitive flexibility in social settings.

Social learning paradigms could offer a nuanced perspective on how individuals update their preexisting expectations based on social feedback. Tasks that measure social learning, how people learn from or about others, typically quantify the extent to which participants shift from initial beliefs and incorporate environmental feedback (Frolichs et al., 2022; Gweon, 2021; Olsson et al., 2020; Rosenblau et al., 2018, 2021, 2023; Sevgi et al., 2020; Wu et al., 2024). These tasks typically use a reinforcement learning framework that quantifies prediction errors – the difference between initial estimates and environmental feedback – and learning rates –how fast or slow participants integrate feedback and thereby adapt to their environment. These metrics pick up participants’ cognitive flexibility – the extent to which participants recognize patterns in the behaviors of others and incorporate them into their decision making.

Prior work suggests that social learning framework can yield transdiagnostic markers of social dysfunction across neuropsychiatric conditions including ASD (Rosenblau et al., 2023). Emerging evidence individuals with an autism diagnosis infer peers’ preferences by referring to their own preferences. Controls on the other hand used trial-level feedback from the peer and preexisting knowledge about of peer preferences to make their inferences. This finding was corroborated on the neural level. Unlike controls, the ASD group did not show prediction error related activity during the social learning task (Rosenblau et al., 2021). These findings may also speak to a broader difficulty shifting perspectives in order to best represent a peer. In a similar vein, individuals higher in autistic traits learned less from their peers during observational learning. Instead of emulating, or representing a peer’s goal, they simply imitated the peer’s action (Wu et al., 2024). Thus, when learning from or about peers, individuals with ASD may have more difficulty shifting beyond their subjective perspective to represent those of others.

In social learning contexts, cognitive rigidity can be conceptualized as an over-reliance on fixed representations. In a study on social learning, autistic adolescents have been found to rely on their own preferences, instead of considering feedback about the person in question (Rosenblau et al., 2021). This can be interpreted as rigidity – sticking with notions about themselves instead of incorporating new and task-relevant information about the other.

To summarize, social cognition is inextricably linked to cognitive flexibility, and there is strong evidence that the social deficits of individuals with autism stem from rigidly sticking to preexisting notions and a diminished adaptation or integration of social cues that would prescribe updating these prior beliefs. From a practical standpoint, cognitive flexibility has been shown to predict adaptive socialization skills. Therefore, interventions targeting domain-general rigidity could aid in improving socialization in autism (Bertollo et al., 2020; Christ et al., 2017; Kenworthy et al., 2014).

#### 4.3.1 Language and communication

Expression and comprehension of language, be it in text or speech, relies on phonological, semantic, syntactic, morphological, and pragmatic knowledge. Cognitive flexibility supports shifting between different linguistic features, such as spelling to sound mappings or semantic senses, to extract meaning (Colé et al., 2014; Deák, 2003; Nation & Norbury, 2005; Swinney, 1981; Treiman et al., 2003). Cognitive flexibility is a crucially implicated in both language development and reading comprehension alike (Colé et al., 2014; Deák, 2003; Ouerchefani et al., 2024; Swinney, 1981).

Language impairments in autism are very heterogeneous. Some individuals are fully nonverbal, while other individuals exhibit domain-specific, above-average verbal abilities (Norbury & Nation, 2011; Saldaña, 2023; Welsh et al., 1987). Notably, studies have consistently reported difficulties with expressive and receptive language as a characteristic feature in ASD. Respectively, expressive and receptive language refer to the ability to produce meaningful language output and interpret language input (Kjelgaard & Tager-Flusberg, 2001; Kwok et al., 2015). Additionally, several studies show evidence of below-average reading comprehension in autism (Jones et al., 2009; Nation et al., 2006; Nation & Norbury, 2005; Norbury & Nation, 2011). Importantly, both reading comprehension and expressive-receptive language are supported by cognitive flexibility.

With regards to reading comprehension, word decoding is a critical prerequisite and relies on the ability to shift between spelling-sound mappings and semantic knowledge. Particularly in nontransparent languages, such as English, wherein spelling-sound mappings are not always consistent (i.e., “yacht”), semantic knowledge can supplement word decoding. Semantic knowledge may include representations of imageability, or how easily a word invokes the sensory representations (i.e., “buoy” vs. “niche”), or frequency, or how often words are used (i.e., “schedule” vs. “panacea”; Ellis, 2002; Graves et al., 2010; Plaut, 1996; Seidenberg & McClelland, 1989; Strain et al., 1995a; Strain & Herdman, 1999).

As indicated by neuroimaging studies, words with inconsistent spelling-sound mappings engage both the visual word form area – implicated in spelling-sound mappings – and a temporoparietal network of regions – such as the inferior temporal sulcus and precuneus cortex – that are relevant for extracting semantic meaning (Graves et al., 2010, 2019). Parietal regions such as the angular gyrus and precuneus have been strongly implicated in maintaining attention and flexibly switching focus, for example, in task-switching paradigms (Corbetta & Shulman, 2002; Dajani & Uddin, 2015; Seghier, 2013; Wager et al., 2005). The overlap in these regions implicated in both reading aloud and cognitive flexibility may signify that word decoding necessitates flexibly shifting between representations of learned spelling-sound mappings and broader semantic representations. Importantly, accumulating representations of spelling-sound regularities and semantic relationships relies on statistical learning (Graves et al., 2010; Strain et al., 1995b; Zorzi et al., 1998).

Decoding words based specifically on spelling-sound mappings can actually be a relative strength for some individuals with an autism diagnosis, and this is reflected in the high incidence of hyperlexia in autism (U. Frith & Snowling, 1983; Huemer & Mann, 2010; Nation et al., 2006; Newman et al., 2007; Norbury & Nation, 2011). In fact, individuals with an autism diagnosis can display comparable, and even enhanced, pseudoword decoding abilities compared to controls and even compared to word decoding (pseudowords are meaningless letter strings that mimic real spelling-sound mappings; Newman et al., 2007; Norbury & Nation, 2011). Yet, reading comprehension in autism lags behind decoding (Norbury & Nation, 2011; Welsh et al., 1987; Zuccarello et al., 2015). Critically, pseudowords are decoded strictly based upon spelling-sound mappings since pseudowords do not have semantic meaning.

In contrast, real words are context dependent and benefit from semantic knowledge (Graves et al., 2010; Strain et al., 1995a; J. S. H. Taylor et al., 2013). Some studies suggest that individuals with ASD may show more inconsistent decoding performance for real words as opposed to pseudowords, and worse performance when decoding irregular words that benefit from semantic knowledge to resolve ambiguities in pronunciation (McCabe et al., 2025; Norbury & Nation, 2011; Zuccarello et al., 2015). Decoding irregular words has been shown to be a crucial aspect for overall reading comprehension (Nation & Snowling, 1998), but more research is needed to specify the relationship between cognitive flexibility and reading comprehension in autism. Reading comprehension difficulties in autism may also stem from broader difficulties with accessing and shifting between different meanings based on contextual information, which is particularly important for pragmatics.

Pragmatics (i.e., figurative language, idioms, puns, sarcasm), especially, rely on broader contextual information and knowledge of what others know and don’t know. Meaning cannot be extracted from the literal semantics of the phrase or words themselves (Gibbs & Colston, 2012; Glucksberg & McGlone, 2001; Ouerchefani et al., 2024). To understand the meaning of a pragmatic phrase, one may need to account for the multiple word meanings, also called polysemy. Polysemic words may refer to concrete or abstract concepts depending on the context (i.e., “heart” might refer to the organ or compassion or dedication; Frege, 1892; Gries, 2019). Additionally, prosody, or how something is said, can serve as a cue for uncovering meaning (“This is ok” with a higher pitch at the end might indicate a question rather than statement; DePape et al., 2012; McCann & Peppé, 2003; Rosenblau et al., 2017; Rutherford et al., 2002). Pragmatics necessitate switching from literal interpretation to accounting for broader linguistic knowledge structures and extralinguistic cues. In turn, cognitive flexibility has been strongly linked to correctly interpreting pragmatic phrases (Kissine, 2012; Ouerchefani et al., 2024; Williams et al., 2023).

Difficulties with use and interpretation of pragmatic language comprise key aspects of expressive-receptive language impairments in autism (Costescu et al., 2022; Kissine, 2012; Kwok et al., 2015). Individuals with ASD have more difficulty with polysemy (Floyd et al., 2021), and tend to use and interpret more literal senses of words and phrases (Graves et al., 2022; Lampri et al., 2024). Additionally, individuals with ASD have more difficulty interpreting extralinguistic cues, like prosody (Costescu et al., 2022; Rosenblau et al., 2017). Each of these elements comprises critical aspects of pragmatic language. Importantly, cognitive flexibility is predictive of the ability to interpret pragmatic language in individuals with ASD (Kissine, 2012; Mashal & Kasirer, 2011). In sum, individuals with ASD may have more difficulties shifting from rote and concrete interpretation of words and phrases to deriving meaning from abstract senses, context, and extralinguistic cues. Thus, pragmatics may contribute to the documented difficulties with expressive-receptive language in autism.

Ultimately, language processing and comprehension arise from representing and integrating several linguistic elements and broader context, which involves cognitive flexibility. More research is needed to understand the relationship between cognitive flexibility in certain language domains and social communication difficulties as assessed in measures of autistic traits and symptoms.

## 5. Leveraging latent dimensions to better specify autism phenotypes

Growing evidence suggests the cognitive rigidity-flexibility dimension represents a transdiagnostic marker for psychopathology (Morris & Mansell, 2018). While the presentation and consequences of cognitive rigidity vary across disorders, the level of cognitive rigidity impacts symptom severity (Meiran et al., 2011; Morris & Mansell, 2018; Rosenblau et al., 2023). In depression, this could manifest as excessively negative views of the world. In certain personality disorders, it may be holding on to negatively biased perspectives of oneself and close others. Stereotypical and compulsive behaviors may be the most striking forms of rigidity in autism or obsessive compulsive disorder (Meiran et al., 2011; Morris & Mansell, 2018; Rosenblau et al., 2023).

In ASD particularly, cognitive rigidity underlies most of the core diagnostic symptom domains (Geurts et al., 2009). More research is needed, however, to investigate how cognitive flexibility in experimental settings relates to behavioral (in)flexibility across the subdomains of autistic traits and in autism symptom clusters. Additionally, future work should aim to define how potential subcomponents of cognitive flexibility predict adaptive living skills in ASD. Cognitive flexibility can be construed as a continuous emergent property describing the adaptability of cognitive systems across subdomains (Braem & Egner, 2018; Dajani & Uddin, 2015; Ionescu, 2012). However, cognitive flexibility could be decomposed into hierarchical subcomponents (Bunge & Zelazo, 2006). For example, set-shifting paradigms may tap into a different aspect of cognitive flexibility than more effortful cued task-switching or reversal learning paradigms (Bunge & Zelazo, 2006; Dajani & Uddin, 2015; Monni et al., 2023).

In order to determine if there are functionally distinct subcomponents which capture differences in the autistic phenotype, we suggest implementing an experimental task battery with paradigms that probe *the cognitive flexibility-rigidity dimension* in relevant symptom domains (see Box 1 for specific domains and possible tasks, also see Figure 1, Panel C). In turn, multivariate dimensionality reduction techniques could help derive the important subcomponents which are relevant to core autistic features and could be applied towards clustering people. This framework is intended to supplement current diagnostic procedures and facilitate data-driven approaches to defining the latent structure of ASD. Such assessment frameworks have been successfully used in athletics research in order to probe common areas of strength across athletes, or, in other words, common factors of athletic competence across various athletic domains. A range of sensory and cognitive assays probe visual perception, higher level cognition, sensorimotor learning, and attention across athletes in various disciplines. These batteries have been shown to successfully distill common areas of strength that are generally relevant for athletes independent of the disciplines, such as visuomotor control or peripheral acuity (Erickson, 2021; Krasich et al., 2016).

Below we list measures that have been shown to sensitively capture cognitive flexibility in key cognitive domains that have symptom areas like perception and attention, language, learning, and social cognition. This framework could be leveraged towards understanding the latent structure of cognitive flexibility – its separable subcomponents – and their role in autism symptomatology.

- **Visual attention and perception tasks**: Tasks probing attentional engagement or coordination between top-down and bottom-up processing. Measuring the ability to spontaneously reverse ambiguous figure illusions could test the coordination of top-down and bottom-up processes on overcoming perceptual biases (Sobel et al., 2005; M. C. Wimmer et al., 2011). Serial visual search tasks, where participants must find a unique shape among distractors and report the orientation of a bar (Li & Theeuwes, 2020), could be implemented to objectively measure attention to detail, which is a factor in autistic traits self-report measures such as the AQ.
- **Language processing**: Deficits in cognitive flexibility can manifest as a difficulty shifting between linguistic elements to derive meaning, resulting in downstream effects on expressive and receptive language and reading comprehension. The Test of Pragmatic Language (Phelps-Terasaki & Phelps-Gunn, 2007) could show how individuals shift to applying contextual knowledge and extralinguistic cues to understanding nonliteral language. The Woodcock Johnson Tests of Achievement (Schrank & Wendling, 2018) include word and pseudoword reading sections. A gap in performance between word and pseudoword reading might signify more rigid use of spelling-sound mappings.
- **Reversal learning**: Associative or reversal learning paradigms that incorporate rewards or social context could probe the nuanced role of cognitive rigidity in learning in social versus nonsocial contexts. Specifically, a candidate task could involve making investment decisions with or without the advice of a confederate of uncertain trustworthiness (Behrens et al., 2008). In these tasks, through associative learning, participants build representations of the confederate’s trustworthiness or likelihood of receiving a return on their investment. To test how participants flexibly adapt their representations to a reversal, the quality of advice from the confederate could change suddenly or the risk of investment could suddenly increase or decrease.
- **Social cognition**: Social learning frameworks, such as learning from or about peers, can quantify cognitive flexibility and explore the latent cognitive mechanisms that govern social learning (Rosenblau et al., 2023; Wu et al., 2024). Importantly, these tasks quantify cognitive flexibility directly by measuring how much individuals incorporate environmental feedback through prediction errors, or how much they represent knowledge or the perspectives of others when it deviates from their own point of view. These finer-grained measures of social learning may better predict social learning difficulties in daily life.
- **Behavioral flexibility in daily life**: Self – and informant – reports and diagnostic assessments can measure convergent validity between individual experiences of difficulties and those of others in daily life. Importantly, we recommend using assessments that have been validated in both diagnosed and undiagnosed populations such as the Vineland Adaptive Behaviors Scale (Klin et al., 2007), which assesses adaptive functioning in daily living, socialization, and communication and the Social Responsiveness Scale (Constantino, 2013) which measures autism symptoms in daily interactions. These assessments can be supplemented with questionnaires that focus on executive functions such as the Broad Inventory of Executive Functions (Gioia et al., 2000), and the Repetitive Behaviors Scale (Bodfish et al., 2000), which probes “insistence on sameness.” Experimental measures of cognitive flexibility in subdomains, as described above, should be related back to self- and/or informant reports of autistic traits and autism symptoms in daily life, in order to test their predictive validity (See Figure 1, Panel D).

Cognitive flexibility can be measured objectively across these domains regardless of diagnostic status of individuals. Moreover, *the cognitive rigidity-flexibility dimension* can be leveraged to develop a framework for examining the latent structure of autism, and potentially distilling subclassifications. English and colleagues (2020) identified social ability, communication, and attention to detail as factors of the autism phenotype. In our model, these could be conceptualized as sub-dimensions which can be measured via experimental paradigms based in neuropsychological research to distill more specific subgroups (see Fig. 1). We propose this approach as a means to improve upon observation-based assessments, which may not detect autism in individuals diverging from prototypical presentations (D’Mello et al., 2022; Harrison et al., 2017; Hiller et al., 2016; Loomes et al., 2017). The presentation of autism in older adults, autistic females, and nonwhite individuals historically fall outside the rigid mold of observation-based criteria for autism (Harrison et al., 2017; Hiller et al., 2016; Walsh et al., 2021). Expanding assessment frameworks to account for a dimensional conceptualization of autism may clarify whether individuals missed by traditional diagnostic techniques fall into certain subtypes. Importantly, while the *cognitive rigidity-flexibility dimension* plays a critical role in autism symptomatology, it is one of multiple candidates which must be examined in a similar fashion.

In summary, though likely not the only higher-order dimension, *the cognitive rigidity-flexibility dimension* governs symptom presentations across different neuropsychiatric conditions, including autism, and can be (Mostert-Kerckhoffs et al., 2015) objectively measured within different subdomains relevant to autism (Fig 1). Rather than building assessment frameworks around a unidimensional presentation of autism that rely on observation and self-report, we propose reframing assessment batteries around broadly applicable higher-order cognitive dimensions, like cognitive flexibility. Specific assessments can then narrow focus in specific subdomains that are underpinned by the higher-order dimension to potentially discriminate between different subtypes. Ultimately, this approach can be a step towards elucidating the structure of ASD, and other heterogeneous conditions, and improving diagnostic specificity such that more individuals acquiring a diagnosis can receive tailored interventions that promote better adaptive outcomes.

## 6. Conclusion

Here we have discussed the need to widen our unidimensional view of autism, in order to better understand less prototypical presentations of ASD and individual differences in autistic traits and autism symptoms (D’Mello et al., 2022; Harrison et al., 2017; Hiller et al., 2016; Walsh et al., 2021). Framing autism or autistic traits as a unidimensional continuum obscures the latent structure of the autism phenotype, both in the general population and in individuals with an ASD diagnosis. It is unclear whether autistic traits are less severe presentations of autism symptoms or a collection of individual differences that are unrelated to the diagnostic criteria for autism (Mottron & Bzdok, 2020). In order to specify the convergence of autistic traits and autism symptoms across populations, we have to study autistic traits and symptoms in conjunction with experimental measures that probe the relevant cognitive functions. Here we focused on one transdiagnostic dimension that holds promise for stratifying the autism spectrum. Specifically, the cognitive rigidity-flexibility dimension can be distilled into measurable subcomponents relevant to the autism phenotype. This dimension can be used to both trace subgroups and clarify whether the autistic traits in the general population measures the same construct as autism symptom severity in individuals diagnosed with autism. This approach can help to specify autism phenotypes towards a multidimensional classification of autism and other neurodevelopmental disorders based on transdiagnostic symptom dimensions. Ultimately, this framework can be used to both improve diagnostic assessments and design more targeted interventions for autism.

## Data Availability

None.

## Footnotes

1 Throughout the text, we refer to individuals holding a diagnosis of Autism Spectrum Disorder as “Individuals with a diagnosis” or “individuals with ASD.” Our rationale for this is that some autistic individuals may not hold a diagnosis or only self-identify. Such cases may be due to uncertainties regarding the latent structure of autism, a lack of characterization of specific subtypes or presentations, or systemic barriers to receiving a formal diagnosis. Therefore, we wish to specify when studies have specifically recruited and tested people holding a diagnosis.
